# Management and Implications of Beta-Lactam Allergies

**DOI:** 10.7759/cureus.60281

**Published:** 2024-05-14

**Authors:** Esteban Zavaleta-Monestel, Keyla Webster, Carolina Rojas-Chinchilla, Gabriel Muñoz-Gutierrez, José Pablo Díaz-Madriz

**Affiliations:** 1 Pharmacy, Hospital Clinica Biblica, San Jose, CRI; 2 Pharmacy, Universidad de Ciencias Médicas, San Jose, CRI; 3 Infectious Diseases, Hospital Clinica Biblica, San Jose, CRI

**Keywords:** penicillin allergy, allergic reactions, cross-reactivity, beta lactam antibiotics, hypersensitivity

## Abstract

Beta-lactam antibiotics are essential components in the current antimicrobial treatment strategy, playing a crucial role in ambulatory patients and hospitalized patients. Despite their prominent therapeutic index, the use of beta-lactam can lead to adverse effects, with allergic reactions being the most concerning because of their severity. Additionally, the phenomenon of cross-reactivity may occur among various beta-lactam families, with side chains significantly contributing to immunological recognition, making these structures often responsible for the cross-allergic reactivity of beta-lactams. Tools to assess beta-lactam allergy include taking a patient's medical history, performing skin tests, and conducting provocation tests. This research aims to analyze the relevant aspects related to the safe administration of beta-lactam antibiotics in hospitalized patients as well as provide knowledge on the proper management of patients with such hypersensitivity, by doing systemic research. This research was made using Google Scholar and keywords such as "Beta-lactam allergy,” "Hypersensitivity,” "Cross-reactivity,” "Desensitization,” and “Beta-lactam allergy management.” In conclusion, substituting a beta-lactam antibiotic with an alternative antibiotic may not always be the best management option for these patients, as it may lead to more adverse effects, be less effective, and prolong hospitalization time. It may also result in higher rates of antibiotic-resistant infections and increased medical costs, as these alternatives are often more expensive. However, an alternative within the beta-lactam family can be sought by conducting the appropriate analyses. Although cross-reactivity does not always occur among all beta-lactams, potential cross-reactivity should always be considered.

## Introduction and background

Beta-lactam antibiotics represent the largest family of antimicrobials and are the most widely used in clinical practice [1]. Their mechanism of action is based on inhibiting the synthesis of the bacterial cell wall by interfering with peptidoglycan synthesis by blocking the final stage of its production, but they also have another mechanism that acts by activating endogenous bacterial autolysin, which destroys peptidoglycan [2].

Beta-lactam antibiotics are crucial in the current antimicrobial treatments, serving a fundamental role for both ambulatory and hospitalized patients. This antibiotic family consists of four distinct groups: penicillins, cephalosporins, carbapenems, and monobactams, each with a varied spectrum of activity. The diverse activity spectra of these molecules enable the effective treatment of a wide range of bacterial infections [3]. Beta-lactam antibiotics, despite their effectiveness in treating various infections, can sometimes lead to adverse reactions. Among these reactions, allergic responses are particularly concerning because of their potential severity and impact on the patient's health. In fact, allergic reactions to beta-lactam antibiotics are the most common cause of drug-induced adverse reactions mediated by specific immunological mechanisms [4].

Advancements have been made in the manufacturing process of beta-lactam antibiotics over the past few years. These improvements have led to a decrease in contaminants present in the antibiotics, consequently lowering their allergenic and immunogenic potential. In other words, improvements have been made in how beta-lactam antibiotics are made. These improvements have led to fewer impurities in the antibiotics, which means they are less likely to cause allergies or immune reactions. Despite these advancements, the number of allergic reactions has not decreased proportionally. This phenomenon may be attributed to the increased exposure of patients to these medications and modifications in their chemical structure aimed at broadening their antibiotic spectrum, leading to the emergence of new antigenic elements. In a study conducted in Latin America, 862 patients with a history of hypersensitivity reactions were surveyed, finding that beta-lactam antibiotics accounted for 13.8% of these reactions, while nonsteroidal anti-inflammatory drugs ranked first in incidence [4].

Studies indicate that 10%-12% of the population, particularly women, report allergies to penicillins. However, only 4%-7% of these reports involve severe reactions. Interestingly, between 70% and 90% of patients who claim to be allergic to penicillins have not experienced hypersensitivity reactions, suggesting that they could potentially tolerate penicillins or other beta-lactams. The far-reaching implications of self-reported allergies include the avoidance of beta-lactams, extended hospital stays, increased readmission rates, higher hospital costs, and an elevated risk of infection [1,5,6]. These allergies also necessitate the selection of second-line antibiotics and can lead to Clostridium difficile infections. Therefore, the accurate diagnosis of penicillin allergies is crucial to optimize both outpatient and inpatient care. Additionally, it is important to establish protocols for administering beta-lactams with high allergy potential, such as penicillin, especially in patients who have never been exposed to the medication [6,7].

Additionally, beta-lactam allergies occur when the immune system reacts strongly to these antibiotics because of a hypersensitivity response. Hypersensitivity reactions are classified in four reaction types and beta-lactam antibiotics can cause any of the four types of hypersensitivity reactions. However, it is shown that allergies to beta-lactam antibiotics typically manifest themselves as type I or type IV hypersensitivity reactions, which will be further discussed in this article. In individuals with beta-lactam allergy, minor antigenic determinants generate IgE-specific responses linked to type I allergy, while major antigenic determinants are often linked to milder urticarial reactions. Apart from the core ring structure, beta-lactam antibiotics possess diverse side chains that can also trigger type I allergic responses [8,9]. These reactions are personalized and can vary in severity. Type II hypersensitivities are less severe, but they can be caused by an IgG response to small molecules such as penicillin that become covalently bound to the outside surface of cells. Type III hypersensitivities are characterized by the formation of small, soluble immune complexes comprising antigens and specific IgG. These complexes deposit in the walls of small blood vessels or the alveoli of the lungs, triggering complement activation and an inflammatory response. This inflammatory process can lead to tissue damage and impairment of its function. Type IV hypersensitivities arise from the response of CD4 T cells to foreign protein epitopes or peptides derived from chemically modified human proteins [8,9].

Additionally, the most common drugs causing hypersensitivity reactions include allopurinol, antibiotics such as beta-lactams, sulfonamides, fluoroquinolones, and macrolides, along with nonsteroidal anti-inflammatory drugs, biological drugs such as bevacizumab, and aromatic antiepileptic drugs. In the case of beta-lactam antibiotics, immediate reactions occur within one to six hours after administration and are usually mediated by IgE. They manifest clinically as hives, with or without swelling, and anaphylaxis. The non-immediate or late reactions occur after six hours of drug administration and are mediated by antigen-specific T cells. The clinical presentation ranges from benign rashes to potentially life-threatening severe cutaneous adverse reactions [8-10].

The objective of this review is to delve into the crucial aspects related to the safe administration of beta-lactam antibiotics, the management of patients with hypersensitivity to these medications, and the optimization of the approach to beta-lactam allergy labeling in both inpatient and outpatient settings.

## Review

Material and methods

Selection Criteria and Search Strategy

Descriptive research was conducted, to provide updated information about patients presenting hypersensitivity reactions to beta-lactam antibiotics, cover aspects such as the mechanism of allergic reactions, their main clinical manifestations, diagnostic tests, and the proper management. A compilation of public articles from digital journals and official websites of health-related databases was conducted, including Science Direct, Springer, SciELO, Elsevier, and The Journal of Allergy and Clinical Immunology, among others. The research was made using Google Scholar and keywords such as "Beta-lactam allergy,” "Hypersensitivity,” "Cross-reactivity,” "Desensitization,” and “Beta-lactam allergy management.” A primary search was conducted between February 10, 2024, and March 30, 2024, and a final search was conducted on April 2, 2024.

Results

Article Selection and Study Demographics

**Figure 1:**
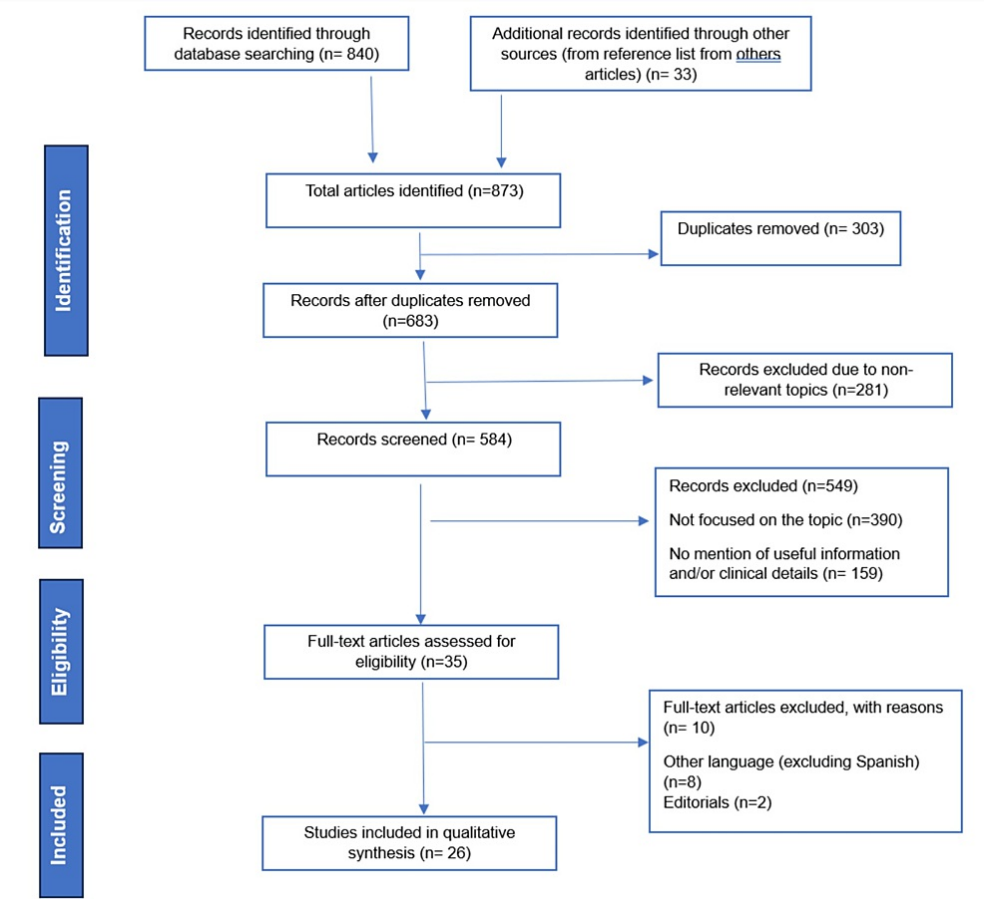
PRISMA flow chart for study selection PRISMA: Preferred Reporting Items for Systematic Reviews and Meta-Analyses

Overall, 873 articles were identified through online database searching. After removing any duplicated entries, 683 articles underwent screening based on their titles and abstracts. Moreover, 281 articles were excluded because of nonrelevant topics. Following this initial screening, a more thorough examination of the full text was conducted for 584 articles. Of the 584 articles, 549 were excluded because of a lack of useful information and clinical details. Furthermore, a total of 35 articles were assessed for eligibility and, finally, a total of 26 articles were included in qualitative synthesis. Figure 1 shows the Preferred Reporting Items for Systematic Reviews and Meta-Analyses (PRISMA) flowchart for the selection of studies.

The characteristics of the included studies are presented in Table 1, which emphasizes the use of beta-lactam antibiotics and the associated hypersensitivity reaction to the antibiotic [1-26]. Six of the mentioned studies were a brief report who showed the results of the allergic reaction of the patients after using certain beta-lactam antibiotics. Four of the mentioned studies were retrospective analyses/studies that compared and showed the result of the allergic reaction of the patients after using certain beta-lactam antibiotics. Besides all of the mentioned studies have discussed the beta-lactam allergy diagnosis tests and their importance. Furthermore, the studies evaluated in these reviews further support that many patients with previously reported beta-lactam allergies, in whom allergy interviews are conducted, can be given beta-lactams with no adverse events noted following administration, and overall stewardship of antibiotics is positively impacted [19-25].

**Table 1:** Characteristics of the included studies

Author name	Year	Journal	Study design	Study setting	Content Summary
Suárez et al. [1]	2009	Enfermedades Infecciosas y Microbiología Clínica	Review	Spain	Beta-lactam classification and chemical structure beta-lactam pharmacokinetics and pharmacodynamics clinical indications for beta-lactam use
Gómez et al. [2]	2015	Servicio de Medicina Interna-Infecciosa	Review	Spain	Indications of beta-lactams in clinical practice
Hamon et al. [3]	2021	EMC - Tratado de Medicina	Review	Unknown	General information about beta-lactam antibiotics, including mechanism, classification, and adverse reactions
Cardona et al. [4]	2021	Revista Alergia Mexico	Review	Mexico	Diagnostic and therapeutic algorithm for non-severe immediate, and severe immediate adverse reactions to beta-lactams. Initial treatment of hypersensitivity reactions to beta-lactam antibiotics
Covington et al. [5]	2019	Pharmacy (Basel)	Review	Switzerland	Conducting antibiotic allergy clarifications
Paño-Pardo et al. [6]	2023	J Investig Allergol Clin Immunol	Guidelines	Spain	Assessment and management of antibiotic allergy labels
Ortega-Cisneros et al. [7]	2022	Revista Alergia Mexico	Review	Mexico	Pathophysiology of penicillin allergy. Cross-reactivity between beta-lactams
Banerji et al. [8]	2023	Journal of Allergy and Clinical Immunology	Review	Unknown	Antibiotic hypersensitivity parameters and characteristics
Jeimy et al. [9]	2020	Allergy, Asthma & Clinical Immunology	Review	Canada	Clinical implications of erroneous labels of beta-lactam allergy. Diagnostic tests for beta-lactam allergy. Alternative antibiotic selection in the setting of confirmed beta-lactam allergy
Bradley et al. [10]	2021	Allergies Journal	Review	United States	Beta-lactam allergy approach and management
Mabilat et al. [11]	2022	JAC-Antimicrobial Resistance	Review	Unknown	Penicillin allergy over-labelling problems and complications
Khan et al. [12]	2022	Journal of Allergy and Clinical Immunology	Retrospective study	Unknown	Classification of drug allergies. Diagnostic tests for drug allergy
Jacobs et al. [13]	2023	Antimicrobial Stewardship & Healthcare Epidemiology	Retrospective analysis	United Kingdom	A retrospective analysis of a beta-lactam allergy assessment that was used in two large hospitals in western Pennsylvania between November 2017 and July 2021. Beta-lactam allergy assessment protocol
Espinola et al. [14]	2020	Archivos de Alergia e Inmunología Clínica	Review	Paraguay	Pathogenesis and pathophysiology of hypersensitivity reaction to penicillins. The allergens arising from the beta-lactam nucleus
Caruso et al. [15]	2021	Journal of Asthma and Allergy	Retrospective study	Unknown	Alternative selection of beta-lactams, IgE-mediated hypersensitivity, T cell-mediated hypersensitivity
Trubiano et al. [16]	2017	Journal of Allergy and Clinical Immunology	Retrospective analysis	Canada	Cross-reactivity and cross-checking: the importance of side chains
Montañez et al. [17]	2015	Drug Allergy Journal	Review	Unknown	Antigenic determinants of beta-lactam allergy. Beta lactam immunological recognition. Alternative treatments for beta-lactam allergy
Blumenthal et al. [18]	2019	The Lancet	Retrospective study	United States	Classification of on-target and off-target adverse drug reactions. Treatment algorithm for patients with penicillin allergy histories
Guzmán et al. [20]	2004	Revista Chilena de Infectología	Retrospective analysis	Chile	Cross-reactivity between penicillins and cephalosporins. Prick test and intradermal reactions in the diagnosis of allergy to beta-lactams
Barberán et al. [21]	2008	Revista Española de Quimioterapia	Review	Spain	Beta-lactam cross-reactivity. Diagnostic and therapeutic algorithm in patients with adverse reaction to a beta-lactam antibiotic
Jaen et al. [22]	2022	El Libro de las Enfermedades Alérgicas	Review	Spain	The most typical antibiotics allergy manifestations. What should be done if there is suspicion of experiencing an allergic reaction to an antibiotic
Ramsey et al. [23]	2023	Annals of Allergy, Asthma and Immunology	Review	Unknown	Studies supporting direct challenges to penicillin in adults
Daghfous et al. [24]	2023	International Journal of Immunopathology and Pharmacology	Retrospective study	Tunisia	Clinical manifestations and time of their appearance according to the tested antibiotic. Different beta-lactam oral provocation test protocols
de Plessis et al. [26]	2019	Journal of Antimicrobial Chemotherapy	Prospective interventional study	United Kingdom	Demographic data and details of reported allergy for the study’s enrolled patients

Discussion

The term “drug allergy” refers to a specific immune response, where the drug acts as a hapten, and the immune response is directed against a hapten-carrier complex that acts as an allergen [8,9]. Allergies to beta-lactams originate from an adaptive immune system response that triggers hypersensitivity reactions. Additionally, hypersensitivity reactions to penicillin may occur without recognized previous exposure and might be because of environmental exposure from animal-origin food such as milk or fungus-producing penicillin [10,11]. Furthermore, hypersensitivity reactions are classified into four types, which are described in the following table (Table 2).

**Table 2:** Classification of hypersensitivity reactions

Hypersensitivity type	Reaction type	Time of onset
Type I	IgE-mediated	Within one hour
Type II	Non-IgE-mediated, cytotoxic	Several hours to days
Type III	Non-IgE-mediated, immune complex	Seven days to 21 days
Type IV	Non-IgE-mediated, cell-mediated	Days to weeks

Mechanism of Beta-Lactam Allergies

Allergies to beta-lactam antibiotics typically manifest themselves as type I or type IV hypersensitivity reactions. In type I reactions, allergens interact with allergen-specific IgE bound to mast cells, basophils, and eosinophils, triggering the release of histamine and other inflammatory mediators. This leads to increased vascular permeability and generalized smooth muscle contraction, potentially resulting in anaphylactic shock. These reactions are immediate and may present with symptoms such as urticaria, redness, breathing difficulties, bronchospasm, swelling, hypotension, tachycardia, altered mental state, or gastrointestinal discomfort [10].

When a beta-lactam antibiotic is administered, the common core ring structure transforms into different antigenic determinants, such as penicilloyl as the primary one, and secondary determinants such as penicillin, penicilloate, and penilloate. In patients with beta-lactam allergy, secondary antigenic determinants trigger specific IgE responses associated with type I allergies. The primary determinant is generally related to less severe urticarial reactions. Besides the structure of the central ring, beta-lactam antibiotics have different side chains that can also cause type I allergic reactions. These reactions are individualized and vary in severity [10,12].

Type IV hypersensitivities are triggered by the activation of CD4 T cells in response to epitopes of foreign proteins or peptides derived from chemical modifications in human proteins. These reactions typically manifest on the skin, presenting as a maculopapular rash or urticaria. Rarely, severe reactions such as Stevens-Johnson syndrome, toxic epidermal necrolysis, or drug reactions with eosinophilia and systemic symptoms may occur because of this hypersensitivity. Type IV reactions have a delayed onset, manifesting several days or weeks after the last exposure, and have also been associated with allergy to beta-lactam antibiotics [10,13].

Clinical Manifestations

The clinical manifestations associated with beta-lactam antibiotic allergy reactions include itching, redness, urticaria, angioedema, and bronchospasm (i.e., wheezing, chest tightness, difficulty breathing, and dry cough). Additionally, laryngeal edema (i.e., throat tightness or dysphonia), hypotension, and abdominal discomfort such as cramps, nausea, vomiting, or diarrhea may occur. On the other hand, more severe reactions can occur because of beta-lactam allergy. Severe reactions encompass cases such as anaphylaxis, drug rash with eosinophilia and systemic symptoms (DRESS) syndrome, Stevens-Johnson syndrome, and toxic epidermal necrolysis [14].

Cross-Reactivity

Cross-reactivity occurs when an individual is hypersensitive to a specific type of medication and develops hypersensitivity to more types of medications within the same family. In the case of beta-lactam antibiotics, the beta-lactam ring, thiazolidine/dihydrothiazine rings, and side chains can sensitize individuals treated with these drugs. Particularly, the side chains significantly contribute to immunological recognition, and, therefore, these structures are often the most responsible for the cross-allergic reaction of beta-lactams [15,16].

Initially, it was thought that all beta-lactams, by sharing a four-membered ring, could provoke cross-reactions, so the classical recommendation was for allergic patients to avoid all beta-lactams. However, subsequent research revealed that some individuals react selectively to a single beta-lactam and tolerate others. It was demonstrated that this occurrence was more common than initially thought, which has led to a change in the original recommendations, which refers to completely avoiding all beta-lactam antibiotics [17].

**Figure 2:**
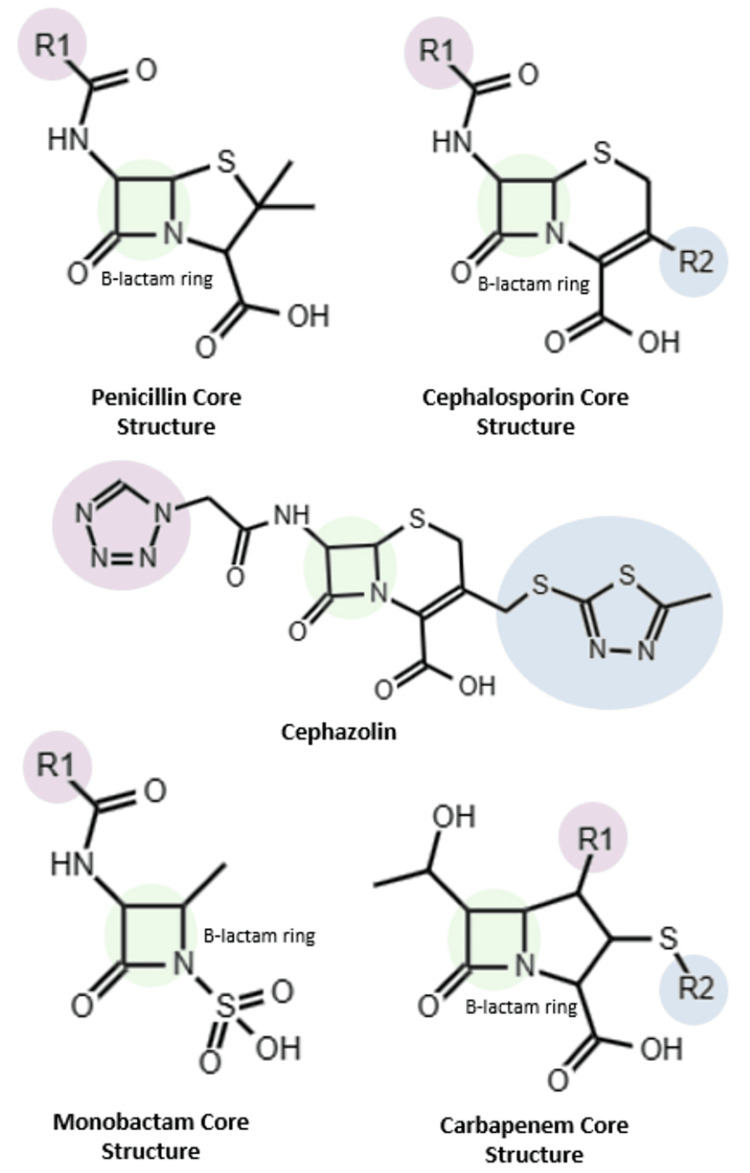
Structural representation of beta-lactam and monobactam antibiotics The varying "R1" and "R2" labels indicate distinct side chains within the spectrum of antibiotics.

Cephalosporins and penicillins share a common structural feature known as the beta-lactam ring. In cephalosporins, this ring is linked to a six-membered dihydrothiazine ring, whereas in penicillins, it is attached to a five-membered thiazolidine ring. Additionally, cephalosporins undergo modifications in the R1 and R2 side chains, a process that broadens their spectrum of activity and enhances their pharmacokinetic and pharmacodynamic properties [6]. These modifications may vary depending on the generation of cephalosporins, with alterations occurring either in R1 alone or in both R1 and R2 (Figure 2).

While there have been numerous reports of allergies to both penicillin and cephalosporins, studies have demonstrated that the actual rate of cross-reactivity is less than 1%. Instances of cross-reactivity are often associated with similarities in the R chain between cephalosporins and penicillins. Notably, cephazolin, a first-generation cephalosporin, does not share a chain with any other beta-lactam compound (Figure 2). Consequently, it is considered safe for use in patients with a history of penicillin anaphylaxis, as per guidelines, without the necessity for testing or additional precautions. Similarly, it is deemed safe for patients with an unconfirmed history of non-anaphylactic reactions to penicillins, eliminating the need for further testing or precautions [26].

Third- and fourth-generation cephalosporins are known to be well-tolerated by patients allergic to penicillin, possibly because their chemical structures have greater differences compared to first-generation penicillins. Additionally, in patients with IgE-mediated allergic reactions to cephalosporins, penicillins may be a treatment alternative in up to 75% of cases [17-19].

Carbapenems have a high probability of developing cross-reactivity with other beta-lactams, and, therefore, they should be avoided in cases of penicillin allergy. This cross-reactivity is likely associated with a response directed toward minor determinants. Monobactams, with their main representative being aztreonam, are composed of a monocyclic beta-lactam ring and a side chain. Although the antigenic determinants produced are analogous to those of other beta-lactams, there have been cases of selective reactions to aztreonam in some individuals, while other beta-lactam allergic patients may tolerate its administration. Furthermore, cross-reactivity between aztreonam and ceftazidime has been observed, as they share the same side chain [20].

Alternative Treatment

Penicillin may serve as a viable option for patients experiencing allergic reactions to cephalosporins, as these responses can sometimes be selective, although there is a potential for cross-reactivity with penicillin. The percentage of patients with IgE-mediated allergic reactions to cephalosporins who tolerate penicillin ranges from 50% to 90%, with variations possibly linked to the specific cephalosporin involved. Tolerance to penicillin tends to be lower with first-generation cephalosporins, likely because of similarities in the chemical structure of the R1 side chain. A recent prospective study involving 98 subjects with IgE-mediated allergic reactions to cephalosporins and positive skin test results confirmed that only 25.5% exhibited cross-reactivity with penicillins [17-19].

The cross-reactivity of cephalosporins has primarily been studied in patients who were initially allergic to penicillins. Following the introduction of cephalothin into the market, cases of anaphylactic reactions were observed in patients with a prior history of penicillin allergy. Initially, the rate of cross-reactivity between penicillin and cephalosporins was overestimated, reaching nearly 50% because of contamination of cephalosporins with traces of penicillin. However, this percentage has gradually declined to 12% since the 1980s [17]. There are varying levels of tolerance observed in individuals with IgE-mediated allergic reactions to cephalosporins when exposed to penicillins; individuals reacting solely to the specific cephalosporin responsible for the reaction and those exhibiting reactions to multiple cephalosporins. The proportion of patients falling into each category fluctuates, likely influenced by factors such as the cephalosporin generation implicated and similarities in side chain configurations. Overall, cephalosporins are generally regarded as a safe option for individuals with IgE-mediated penicillin allergies, with a considerable percentage showing tolerance. However, tolerance levels may decrease if the side chain of the cephalosporin matches that of the initial allergen [17,18].

In the past decade, there has been considerable interest in investigating the extent of cross-reactivity between carbapenems and penicillins in patients with allergic responses. Several prospective studies, primarily conducted by the same research group, have emerged, focusing on both adults and children with IgE-mediated allergic reactions to either penicillins or cephalosporins. Among these studies, two specifically examined patients with IgE-mediated hypersensitivity to penicillin, revealing that a small percentage tested positive for skin reactions to imipenem or meropenem. Notably, all cases that tested negative for skin reactions tolerated the administration of these carbapenems. These findings have been recently reaffirmed through studies involving imipenem, meropenem, and ertapenem. These studies indicate that carbapenems can be a safe alternative in more than 99% of patients with either IgE- or T cell-mediated reactions to penicillins or cephalosporins [17-19].

Diagnostic Tests for Beta-Lactam Allergy

The tools for evaluating beta-lactam allergy include taking a patient's medical history, performing skin tests, and conducting provocation tests. A detailed and accurate medical history is essential as the first step for an accurate diagnosis. The medical history should contain detailed information about the time elapsed since the reaction, the antibiotic associated with the reaction and its purpose, the dose and route of administration of the antibiotic, the signs and symptoms presented by the patient, and, finally, the treatment received during the reaction. The positive predictive value of the medical history for identifying hypersensitivity to beta-lactam antibiotics is quite low (19%), and around 33% of patients with positive results on skin tests have imprecise medical histories [4,21].

Skin tests involve the application of small amounts of the antibiotic or components of its chemical structure to the skin, either by superficial injection (prick test or puncture), dermal injection, or application onto the skin (patch test). Depending on the type and severity of the reaction, a specific testing method will be recommended [22]. Skin tests are useful for analyzing both type I and type IV hypersensitivity reactions; however, clinical experience is required to perform and interpret the results. Although in clinical practice these tests are usually performed after the refractory period, which is six weeks after the reaction (to avoid false negative results), there is little evidence to support this practice, so they could be performed earlier if clinically indicated. Additionally, it is essential to carry out skin tests shortly after the reaction, as only between 20% and 30% of patients with positive skin tests maintain that sensitivity after 10 years [4,21].

If skin tests are negative and there is still suspicion of a hypersensitivity reaction, a provocation test should be performed. Besides, the risk-stratification strategy for direct oral provocation tests can reduce the need for unnecessary skin testing in patients with low-risk penicillin allergy histories. The purpose of this test is to confirm the diagnosis. Provocation tests involve the gradual administration of increasing doses of a medication until one or more therapeutic doses are reached, using single-blind protocols [20,23]. In the case of children and low-risk patients, such as those with isolated nonallergic symptoms such as itching without skin lesions or gastrointestinal symptoms, family history of penicillin allergy, or little-known reactions occurring more than 10 years ago without signs suggestive of an IgE-mediated reaction, an oral provocation test without prior skin testing is recommended. This is because most reactions are nonallergic or related to viral rashes. It is important to consider that provocation tests cannot be performed in those that are unethical because of the severity of the reaction that the patient presented, for example, severe immediate reactions or severe delayed reactions such as Steven-Johnson syndrome, DRESS syndrome, anaphylaxis, and toxic epidermal necrolysis [4,24].

Approach and Management of Allergic Patients

The initial management of patients with a possible reaction to beta-lactams in the acute phase includes conducting a complete medication history, including the type, dose, route of administration, and duration of treatment. Likewise, a detailed description of the patient's symptoms and signs, with a comprehensive examination of the skin and mucous membranes, including the mouth, eyes, and genitals, is necessary. Additionally, signs of danger/severity should be sought, which include clinical symptoms as well as certain laboratory parameters. This approach will lead to a correct diagnosis and the appropriate selection of allergy tests, and this would facilitate the decision of whether to discontinue the medication or not. In the presence of signs of danger/severity, suspected drugs should be stopped immediately [14].

The main treatment for patients with immediate and non-immediate hypersensitivity reactions to antibiotics is to avoid the medication responsible for the reaction and the use of alternative antibiotics. The choice of safe alternative antibiotics is made by analyzing the potential cross-reactivity of beta-lactams, considering the similarity of their side chains. However, some situations significantly restrict alternative treatment options, such as complications associated with patient comorbidities, drug interactions, antibiotic availability and cost, antibiotic coverage, and microbiological resistance profile. In these cases, desensitization is recommended when there is no viable alternative to a beta-lactam antibiotic [4].

Desensitization 

The treatment of drug hypersensitivity reactions in patients who have no other therapeutic alternatives is based on the desensitization process. This procedure induces a temporary state of hyporesponsiveness by gradually administering suboptimal doses of the drug that trigger hypersensitivity until reaching the necessary dose. Notably, once a patient undergoes desensitization, regular and uninterrupted exposure to the medication is required to prevent the process of resensitization. Desensitization protocols are designed for patients who have demonstrated, through skin tests or IgE measurement, an IgE-mediated mechanism against the responsible drug. There are two types of desensitization protocols known, one is rapid desensitization, used in patients with a history of immediate-type adverse reactions, where the mechanism involves mast cells, basophils, and IgE. The second is slow desensitization, commonly used in patients with non-immediate type IV reactions, wherein the mechanism is mediated by T cells [4,9].

Mast cells and basophils play a crucial role in immediate reactions, whether an IgE-mediated mechanism is present or not. It is said that the gradual administration of subtherapeutic doses of the drug during desensitization provides enough antigenic determinants, which bind to the IgE present on the mast cell's high-affinity receptor; however, it does not cross-link with this IgE. This results in a decrease in cellular response, possibly because of the reduction of intracellular calcium mobilization, which is an important factor for degranulation and cytokine production in these cells. Besides mast cells, desensitization appears to impact other cells, as it has been successfully used in T cell-mediated reactions. Thus, desensitization is an antigen-specific process, and the induction of specific drug-reactive regulatory T cells has been observed [4,25,26].

Desensitization enables the physician to prescribe and administer the antimicrobial treatment of choice, which is typically the first line [25]. There are multiple protocols for desensitization to penicillin, with the most well-known involving the progressive administration of increasing doses orally, subcutaneously, or intravenously, starting with dilutions ranging from 1:100 to 1:1000 of the total therapeutic dose. The administered dose is doubled at intervals of 15 to 60 minutes until the therapeutic dose is reached [4]. This procedure can take between four to 12 hours, and desensitization may last up to four half-lives of the medication, after which sensitivity is considered to have returned. As for cephalosporin desensitization, separate evaluations for each cephalosporin are necessary, although the same principle of gradually increasing doses every 15-60 minutes is maintained until the target dose is achieved [4].

## Conclusions

In conclusion, effective and safe antimicrobial treatment hinges on the proper management of allergies to beta-lactam antibiotics. This research underscores the importance of accurately evaluating penicillin allergies before dismissing its use, given that a significant number of self-reported cases do not result in clinically significant reactions. The unwarranted substitution of these antibiotics could precipitate adverse outcomes, including antibiotic resistance and escalating medical costs. Consequently, it becomes crucial to introduce educational initiatives and personalized strategies for managing beta-lactam allergies. Additionally, integrating antimicrobial stewardship programs is key to ensuring that each patient receives optimal and safe treatment.
